# Automated Object Detection in Experimental Data Using Combination of Unsupervised and Supervised Methods

**DOI:** 10.3389/fphys.2022.805161

**Published:** 2022-04-06

**Authors:** Yiran Wu, Zhen Wang, Crystal M. Ripplinger, Daisuke Sato

**Affiliations:** Department of Pharmacology, University of California, Davis, Davis, CA, United States

**Keywords:** machine learning, unsupervised learning, *k*-means clustering, support vector machine, object detection, image processing, artificial intelligence, cardiac images

## Abstract

Deep neural networks (DNN) have shown their success through computer vision tasks such as object detection, classification, and segmentation of image data including clinical and biological data. However, supervised DNNs require a large volume of labeled data to train and great effort to tune hyperparameters. The goal of this study is to segment cardiac images in movie data into objects of interest and a noisy background. This task is one of the essential tasks before statistical analysis of the images. Otherwise, the statistical values such as means, medians, and standard deviations can be erroneous. In this study, we show that the combination of unsupervised and supervised machine learning can automatize this process and find objects of interest accurately. We used the fact that typical clinical/biological data contain only limited kinds of objects. We solve this problem at the pixel level. For example, if there is only one object in an image, there are two types of pixels: object pixels and background pixels. We can expect object pixels and background pixels are quite different and they can be grouped using unsupervised clustering methods. In this study, we used the *k*-means clustering method. After finding object pixels and background pixels using unsupervised clustering methods, we used these pixels as training data for supervised learning. In this study, we used logistic regression and support vector machine. The combination of the unsupervised method and the supervised method can find objects of interest and segment images accurately without predefined thresholds or manually labeled data.

## Introduction

Cardiac cells and tissue have complex shapes. In addition, muscle contraction changes their shapes over time. When we analyze experimental data such as calcium concentration in the cell or membrane potential in tissue, it is necessary to segment out objects of interest from their background. If data contain signals from the outside of objects of interest, statistical values such as means, medians, and standard deviations can be erroneous.

If there are only a few images, it is possible to do it manually. However, for example, movie data contain many frames. In this case, it is desirable to automatize the process. There have been many ways to do it automatically, at least partially. One way is to use a thresholding method if the background is less noisy and contrast between objects of interest and the background is relatively high (Gonzalez and Woods, 2018). However, it is often difficult to choose an appropriate threshold value, especially when the image data contain noisy or weak signal parts. Another way is to use the center of the object, which is confident, instead of using the whole object. However, the center and the fringe may have different signals and the signals from the fringe might be important.

More recently, machine learning, such as deep neural networks (DNNs), has been greatly improved, especially for image processing. DNNs have shown their success through computer vision tasks such as object detection, classification, and segmentation of image data including clinical and biological data. However, supervised DNNs require a large volume of labeled data to train DNNs and tuning of hyperparameters. For example, without segmentation maps as training data, convolution neural networks such as U-net (Ronneberger et al., 2015; Zhou et al., 2018; Khened et al., 2019) cannot be applied to the problem. In addition, we use various tools and materials in research experiments. This means we have to re-tune and re-train the model as we change the experimental settings. The goal of this study is to segment out objects of interest from the background automatically.

In this study, we show that the combination of unsupervised learning and supervised learning can automatize this process and find objects of interest accurately. Instead of DNNs, we used simple machine learning methods that require little time to train and have short inference time. As we show later, we can expect signals from object pixels and signals from background pixels are quite different and thus they can be grouped using unsupervised clustering methods. On the other hand, supervised learning methods often give better results. We proposed a combined method that possesses advantages of these two methods. Here, we used the unsupervised method to extract training data and the supervised method for prediction to find objects of interest accurately without the need for manually labeled data.

## Methods

### Experimental Data Sets

**FIGURE 1:**
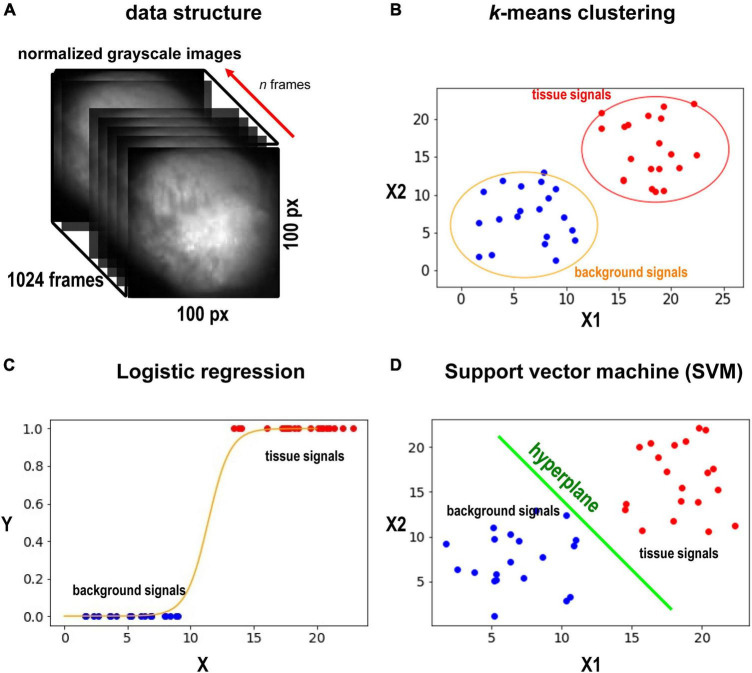
Data sets and methods. **(A)** Grayscale image of the heart. The heart is located in the center of the image. We select *d* frames, and each pixel would have d values (*d*-dimensional vector) from the frames. **(B)** The schematic illustration of the k-means method. Here only two axes are shown. In our case, we compute in *d*-dimensional space. k-means is an unsupervised method, and no training is required. **(C)** The schematic illustration of SVM. SVM will find the best hyperplane to split the data. SVM requires training. **(D)** The schematic illustration of the logistic regression. the logistic regression will fit sigmoid function to the data points and find a middle point (*y* = 0.5) to split the points to the two classes (tissue and background). The logistic regression classifier requires training. We note that **(B–D)** are only for illustration. The actual dimension of the data in this study is *n*.

In this study, we used movie data of action potential wave propagation in the heart. Each movie contains one heart object close to the center of the frame. Cardiac tissue was loaded with a fluorescent indicator (RH237), which changes the recorded fluorescence with changes in membrane potential. The goal here is to segment images into the heart and background in these movies. Data were collected at 1 kHz. Each movie consists of 1,024 frames (= 1,024 ms), while each frame is a 100 *px ×* 100 *px* 8 bit-grayscale image (Figure 1A). Pixel values were normalized between 0 and 1. This was done for each pixel. 0 was assigned to the minimum value of the pixel over time (not the minimum value of the entire movie). Similarly, 1 was assigned to the maximum value of the pixel over time. One data point consists of *n* frames. *n* is arbitrary. In this study, we chose *n* to be 32, 128, 256, 512, and 1,024. For example, when we acquire boundaries of objects for the first frame with *n* = 512, we use from frame 1 to frame 512. Each data point is classified individually. Then, using the result, we create a mask to hide the background for the first frame. Similarly, for the *n-*th frame, we use from frame *n* to frame *n*+511. In general, the larger *n* gives better classification performance. On the other hand, the smaller *n* gives better motion tracking performance. In this study, since the image contains 10,000 pixels, there are 10,000 data points to be classified.

### *k*-Means Clustering

**FIGURE 2:**
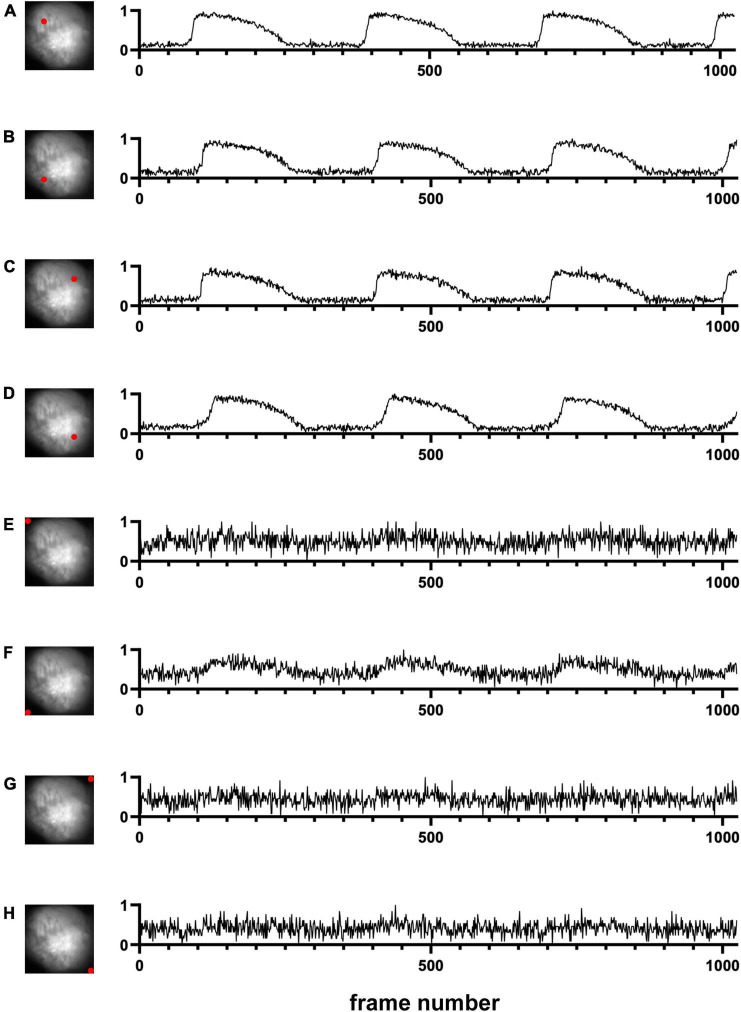
Signals in time domain (Dataset No. 1). **(A–D)** Representative data from four pixel locations from cardiac tissue. **(E–H)** Representative data from four pixel locations from the background. For each plot, the x-axis represents the time, and the y-axis represents the change in values of one pixel location over time. Due to normalization, noise in background pixels is normally amplified **(E–H)**.

We first used one of the classical unsupervised methods called *k*-means clustering (Forgey, 1965; MacQueen, 1967; Hartigan and Wong, 1979). *k*-means clustering is originally used for signal processing that groups *x* data points into *k* clusters. Cardiac cells loaded with a fluorescent indicator show action potential signals and thus we expect the signals from the heart tissue would be different from those from the background in the data space (Figure 1B). We note that the dimension of the data space represented in Figure 1B is two. The dimension of data space in this study is *n*. We sampled pixels from heart tissue and background and plotted the change in values of these pixels over time (Figure 2). Figures 2A–D show representative signals from the heart tissues while Figures 2E–H show representative signals from the background. These panels demonstrate the distinct signal patterns in the object of interest and the background areas. In this study, we use the similarity of time-series data.

### Manually Selected Training Data

In addition to unsupervised machine learning, we also used supervised machine learning which requires training datasets. We manually selected the heart areas from around the center of the heart and non-heart areas from around the four corners. Note that we only selected the regions far from the boundaries to assure the selected pixels belong to these classes. Since in our movies the heart/non-heart area do not change radically over time, our selection of the first frame could be applied to the whole 1,024 frames. The detail of the process is as follows:

1. Draw five polygons, corresponding to the central heart areas and four corner non-heart areas, avoiding the marginal areas.
2. Identify the pixel location circled by these polygons, and extract pixel values from the selected areas of 1,024 frames, respectively.
3. Append heart/non-heart labels (0, 1) to the two types of extracted values to create a dataset of shape (*m*, 1,025*)* (1,024 frames + 1 label), where *m* is the number of selected pixels.

We labeled 5,500 heart and 900 non-heart pixels, that is *m* = 6,400. Our training and validation sets are split with a ratio of 8:1 for both heart and non-heart pixels.

### Logistic Regression

In this study, we used logistic regression and support vector machine (SVM) as supervised learning methods. Logistic regression is one of the simplest yet effective classification methods (Pregibon, 1981; Peng et al., 2002), which models the probability of a solid outcome using logistic function. For a binary logistic regression model, the outcome would be 1/0, true/false, while in our case, it would be heart/background (Figure 1C). In terms of solving the problem, let X be the input pixel value and Y be the label, logistic regression defines a relation of *f*(X) = Sigmoid (m*X + c) (m and c are weights and bias), where Sigmoid (t) = 1/[1+ exp(-t)]. The goal is to minimize [Y-f(X)]^2^. The procedure is as follows:

1. Using the manually selected training data with dimension (6,400, 1,025) from 2.3, select *n* frames (0 < *n* < = 1,024) to prepare a new dataset with new dimension (6,400, *n*+1), including the label.
2. Train the logistic regression model with the selected data. Predict any single frame with input shape (10,000, *n*), and the output shape of (10,000, 1) can be reshaped to *100 px × 100 px* to get the mask.

### Support Vector Machine

We also used SVM as another example of supervised machine leaning. SVM is a robust classification model (Suykens and Vandewalle, 1999). SVM constructs a hyperplane in the data space to split the data points into the two classes (Figure 1D). While we are choosing the hyperplane, we want to maximize the minimal distance between the classes. When we draw a hyperplane to split the two classes, each class would have a point that have the minimal distance to the plane, and what SVM tries to do is to maximize this distance. We used the linear kernel for all the cases. The procedure is similar to logistic regression in section “Logistic Regression,” except we train SVM in step 2.

### Combined Method of *k*-Means Clustering and Support Vector Machine

As we will show in the “Results” section, SVM gives the most stable and accurate results within these three methods (*k*-means clustering, logistic regression, SVM). However, SVM requires manually labeled training data. On the other hand, with unsupervised machine learning, *k*-means does not work as well as SVM, but it does not require training data. Thus, it is natural to combine these two methods for one that performs well while does not need labeled training data. We first modify *k*-means to retrieve data points for training, and then applied SVM using those training data.

We first randomly choose a center for each cluster for *k*-means clustering, and then assign each data point to the nearest cluster. In our case, we have two clusters: heart tissue pixels and background (non-heart) pixels. Since we are using *k*-means to prepare data for training, we want to avoid the marginal areas and select the confident areas, just like when we select manually. We proposed *Distance Discount Factor* to help selecting the confident areas in *k* -means clustering.

*Distance Discount Factor in k-means clustering* is as follows. Let a data point A have a distance D_1_ to Center 1 and D_2_ to Center 2. We compare D_1_ and D_2_ to decide to which cluster A belongs. During this step, we multiply distance D_1_ with a discount factor γ ∈ (0,1] to make D_1_ smaller, that points are more likely to be assigned to Center 1 with the discounted distances. Despite the discount factor, there would still be points assigned to Center 2 with D_2_ < γD_1_. These points would be viewed as stubborn points; they are so close to Center 2 that they cling to that center even after we posed a discount to D_1_. Then these stubborn points would be the confident points that belong to this cluster, which would be extracted and labeled as training data. Despite we designed γ here to be in range (0,1] for convenience, a more loose range would be γ ∈ (0,∞). Since we have inequity “D_2_ < γD_1_,” multiply one side by γ would be the same to multiply another side by 1/γ.

The basic algorithm of *k*-means clustering is not changed with the discount factor. At each iteration of *k*-means clustering, the discount factor would be applied when comparing distances of each point to the two centroids, which is basically a metric to decide how the points would be assigned to each centroid. With discount factor = 1, this is just the original k-means since the distances would not be changed, while with discount factor = 0, all points would be assigned to one cluster, since we assign points to the nearest centroid but distances of all points to this cluster are multiplied by 0.

The procedure of the combined method is as follows:

Input: A movie consisting of 1,024 frames, while each frame is a *100 px × 100 px* 8 bit-grayscale image. Thus, there are 10,000 (× 1,024) data points.

1. Run k-means with the distance discount factor twice (one for the tissue and one for the background) on these data points to find confident heart tissue points (Figure 4B) and confident background points (Figure 4C), substituting the step where we manually select data. With these confident points we form a dataset of *m × 1,024* and corresponding labels of *m × 1* (let *m* be the number of confident points). Note that the discount factor needs to be tuned with visual observation only once for a specific task, in our experiments we tuned the discount factor with grid search on Dataset No.1 and it can be used for other datasets.
2. Set *n* to be the number of frames we extract. To segment the *i* th frame (*n, i* ∈ [1, 1,024]*; n + i – 1 < = 1,024*), we extract frame *i* ∼ frame *n*+ *i – 1* and form a training dataset of *m ×* (*n*+ *1*), with *“+ 1”* representing the labels.
3. Fit SVM (or other supervised methods) to this labeled dataset to get a classifier. Then for the *i* th frame, we use this classifier to classify each of the 10,000 pixels and get segments.

### Noise Reduction With Modified Median Filter

We sometimes observed dotted noise in regions where signals are obscure. At the final stage, we applied a modified median filter to reduce noise specifically in masks (with only 0s and 1s) (Supplementary Figure 6). This function is similar to original median filter, but it is more flexible and is tailored for masks.

After classification, each pixel in the mask has a value of either 1 (heart pixel) or 0 (non-heart pixel). For each pixel location, we check whether its neighbor’s value is the same as itself. We count these occurrences to determine whether to change its value. For example, assuming a pixel A has a value 1, we count the 3 × 3 neighbor pixels surrounding A. With a threshold of 3, if more than 3 pixels in the grid have the value of 1, we kept A unchanged. Otherwise, we change A to 0 since its neighbor’s counts does not satisfy the threshold.

We make the function more flexible by enabling it to be applied to only one value. That is, if we set remove heart to false, the counting grid will not be applied if the pixel has a value 1 (heart pixel). Then the 1 s in the mask remain intact, while the 0 s can be changed to 1 if it does not satisfy the filter threshold.

### Code Availability

Codes are written in Python and MATLAB and are available from GitHub^1^.

## Results

In this study, we detected the heart in the experimental image data using unsupervised and supervised machine learning methods. We used *k-*means clustering as an unsupervised method, and logistic regression and SVM as supervised methods.

**FIGURE 3:**
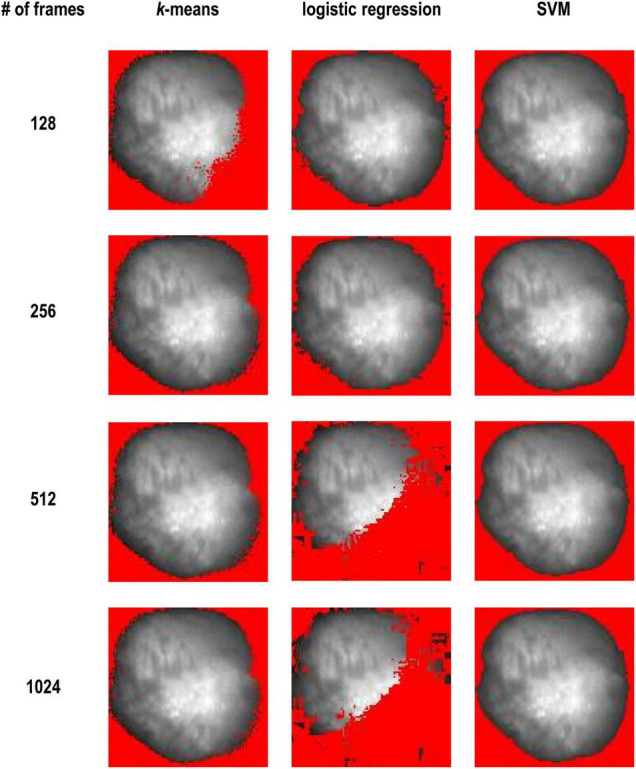
K-means vs. logistic regression vs. SVM and with 128, 256, 512, and 1,024 frames (Dataset No. 1). The detected background was masked with red and only the heart area is shown.

Figure 3 shows comparison of results between these three methods. *k*-means clustering worked better as the number of frames increased. On the other hand, logistic regression worked well when the number of frames was small. SVM worked well regardless of the number of frames.

**FIGURE 4:**
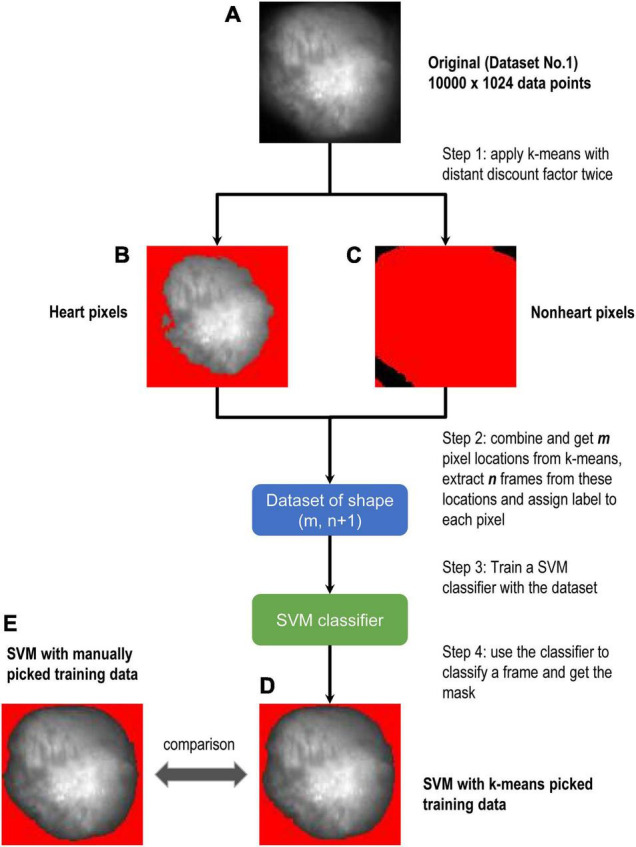
Flow chart of combined method. **(A–E)** Are all from Dataset No.1. *k*-means was used to get the training data at the first step. Border zones were excluded from the training data. We chose data close to the centroids. The non-red area in **(B)** is heart and the non-red area in **(C)** is non-heart part. Then theses selected areas were labeled with 0 and 1 s to train SVM. **(D,E)** Are the results of the SVM model, where SVM of **(D)** is trained by k-means picked data and **(E)** is trained by manually picked data.

Although SVM gave the most stable results among them, it requires training to use it. For Figure 3, we manually selected the training data as described in section “Methods.” As the next step, we considered to prepare training data using unsupervised learning. Figure 4A is the original image data. We applied the *k-*means clustering with the distance discount factor and picked the heart pixels (Figure 4B) and non-heart pixels (Figure 4C). Using training data picked by the *k*-means clustering, we trained the SVM and classified heart and non-heart pixels. Figure 4D is the detected heart. Figure 4E shows the detected heart using manually picked training data for comparison. Here, we used 1,024 frames. We also varied the number of frames and tested the combined method (Supplementary Figure 1). Supplementary Figures 2–5 shows all the results and comparisons between the combined method and SVM using manually selected training data. In all the cases, both results are very similar.

We also combined *k-*means clustering and logistic regression. However, in this case, the results were worse than the combined method of *k-*means clustering and SVM. Supplementary Table 1 shows all the cases we did in this study.

SVM has a validation accuracy of 0.999 for all frames on both manually selected data and *k-*means selected data. Logistic regression has a validation accuracy of 0.999 for 128 and 256 frames, 0.59 for 512 frames, 0.5 for 1,024 frames. We calculated the ratio r = (the number of heart pixels)/(total number of pixels) with different methods (Supplementary Table 1). To get a clearer insight into the change of ratio with respect to the change in frames, we calculated the standard deviation (STD) of the heart ratios from each method (Supplementary Table 1). We expect STD would measure the consistency of each method, that is how the ratio is varied with different number of frames. Our results show SVM is much more consistent (lower STD) than logistic regression with either manually labeled data or *k-*means extracted data. Both methods have a lower STD with *k-*means extracted data than that with manually selected data.

### Moving Objects and Boundaries

This algorithm can be applied to the moving objects and can track moving boundaries. Supplementary Movie 1 shows the result when tissue is rotating. Supplementary Movie 2 shows the result when tissue is shrinking. We also tested this algorithm using calcium transient data along with contraction by Huebsch et al. (2015). In these examples, we chose smaller frame number (*n* = 32). In all the cases, we could track moving boundaries accurately.

## Discussion

In this paper, we proposed the combined method of unsupervised machine learning and supervised machine learning to automatize the process of object detection mainly focusing on the cardiac movie data. We used the fact that most experimental data contain only a few objects and signals from the background are quite different from those of the objects of interest.

Unsupervised methods and supervised methods have their own advantages and combined unsupervised and supervised methods have been used in several areas. For example, Richard et al. (2007) used combined unsupervised and supervised methods for segmentation of radiophonic audio streams. Guo et al. (2015) used them to automatize lesion detection on MRI scans. In this study, we solved the problem of the heart tissue segmentation in grayscale movies. We formulated the problem from the per-pixel level instead of the per-image level. The advantages of the per-pixel view are: 1. Each pixel would be classified and thus the precision of the boundaries would be high. 2. The problem could be viewed as a simple binary classification problem. In one frame of the movie, the 100 × 100 pixels are viewed as 10,000 datapoints, which are reasonably enough for training of the model, and all we need is to classify them as heart or non-heart pixel and form a mask.

Many segmentation methods have been proposed to find objects in an image (Gonzalez and Woods, 2018). However, most of them are for a single image. In this study, we used multiple frames in the movie data to segment out objects of interest. In other words, we do not use spatial similarity. Instead, we use temporal similarity. This makes the algorithm robust for noise. In fact, due to normalization of signals over time, even if the noise levels are the same in the tissue pixels and the background pixels, the noise signals would be amplified in the background pixels (Figures 2A–D vs. Figures 2E–H). Thus, even during diastole, we can classify the tissue pixels and the background pixels.

In this study, we compared various methods. We first used unsupervised and supervised methods individually (Figure 3). We found that unsupervised learning like *k*-means clustering works in most cases, especially when the number of frames is large. *k*-means clustering does not require labeled data or predefined thresholds. However, in some cases, it could not identify the heart properly. *k*-means fails to segment the images when the number of frames is small (Figure 3, 128 frames), but it worked with more than 256 frames. Since change in the signal is small with a smaller number of frames, clustering cannot distinguish these pixels and background pixels where zero signal plus noise. With more data points, change in the signal from heart tissue becomes larger and the classification results became better with 256 frames or more. In other words, the model becomes more robust with higher dimensional data.

SVM performed better than logistic regression and *k*-means clustering. SVM worked with all the number of frames and the results are quite stable (Figure 3), but it requires labeled data to train. Thus, we combined the advantages of *k*-means clustering and SVM.

To choose reliable training data sets, we developed the distance discount factor strategy. The distance discount factor can be between 0 and 1. If the distance discount factor is 1, it is the same as the original k-means clustering. As the distance discount factor becomes smaller, we will find smaller subsets of points close to the centroids. Using this algorithm, we can find more reliable locations of centroids and exclude outlier data points as well as data points near the boundaries.

In this study, we chose the distance discount factor manually so that there would be a reasonable number of training data. But it is easy to automatize the process if the target number of training data is given.

Our results showed that the combined method performed well while it does not require any manual labeling. We confirmed that the combined method has similar results comparing to SVM with manually labeled data in almost all cases (Supplementary Figures 2–5). We also tested this method with a smaller number of frames (32 frames) (Supplementary Figure 7). Thirty-two frames are equal to 32 ms as the data acquisition rate is 1 Hz. In 32 ms, the motion of the heart is limited. Thus, when this method works, we can track the motion of the heart (Supplementary Movies 1, 2).

We imagine that logistic regression would have a better fit for classification problem due to nature of sigmoid function. From the results, logistic regression worked with 128 and 256 frames but failed with 512 and 1,024 frames (Figure 3). We tested with several numbers of frames and found that the effectiveness of the method decreases gradually as the number of frames (i.e., dimension of the data) increases. Regression methods loss its generality with higher dimensional data, as they are unable to draw an unregular line or shapes between the two classes in high dimensional spaces.

Our current task required labels only for one object and thus there were two classes. It is easy to extend the algorithm to increase the number of objects. If there are two types of objects in an image, there are three types of pixels. For a more complicated scenario, the cost of labeling becomes large. In this paper, we experimented with grayscale video data, and we combined *k-*means clustering with SVM to separate background noise from object of interest accurately. There are many other unsupervised and supervised methods. In future studies, we will try other combinations of unsupervised and supervised methods for more complicated scenarios.

## Data Availability Statement

The datasets presented in this study can be found in online repositories. The names of the repository/repositories and accession number(s) can be found below: https://github.com/DSatoLab/Automated-Object-Detection-with-AI.

## Author Contributions

All authors listed have made a substantial, direct, and intellectual contribution to the work, and approved it for publication.

## Conflict of Interest

The authors declare that the research was conducted in the absence of any commercial or financial relationships that could be construed as a potential conflict of interest.

## Publisher’s Note

All claims expressed in this article are solely those of the authors and do not necessarily represent those of their affiliated organizations, or those of the publisher, the editors and the reviewers. Any product that may be evaluated in this article, or claim that may be made by its manufacturer, is not guaranteed or endorsed by the publisher.

## Footnotes

1
https://github.com/DSatoLab/Automated-Object-Detection-with-AI

## Supplementary Material

Supplementary Figure 1 Comparison between trained SVM using data from K-Means and manually selected data (Dataset No. 1).

Supplementary Figure 2 Results of the combined method (Dataset No. 1) with 128 Frames.

Supplementary Figure 3 Results of the combined method (Dataset No. 2) with 128 Frames.

Supplementary Figure 4 Results of the combined method (Dataset No. 3) with 128 Frames.

Supplementary Figure 5 Results of the combined method (Dataset No. 4) with 128 Frames.

Supplementary Figure 6 Comparison between k-means and smoothed k-means with 128, 256, 512, and 1,024 frames (Dataset No. 1). The detected background is masked with red and only the heart area is shown.

Supplementary Figure 7 Results of the combined method (Dataset No. 1) with 32 Frames.

Supplementary Table 1 Ratio of Predicted Heart Pixels with different frames (Dataset No. 1). Ratio the number of heart pixels predicted by each method and different number of frames (Total of 10,000 pixels). STD is the standard deviation of each method (each row) to see the variation of each method with different number of frames. k-means used unlabeled data; Logistic regression and SVM used manually selected data to train; Combined SVM and combined logistic regression used labeled data from k-means clustering.

Supplementary Movie 1 The result of the combined method when tissue was rotated 180 degrees over 1 s. The detected background is masked with red and only the heart area is shown. *n* = 32.

Supplementary Movie 2 The result of the combined method when tissue was shrunk over one second. The detected background is masked with red and only the heart area is shown. *n* = 32.
