# Supplementary material for: Automated Object Detection in Experimental Data Using Combination of Unsupervised and Supervised Methods
Source: Front Physiol. 2022 Apr 6;13:805161. doi: 10.3389/fphys.2022.805161 (PMC9019745; doi:10.3389/fphys.2022.805161)
Supplement: Supplementary Figure 1 — Comparison between trained SVM using data from K-Means and manually selected data (Dataset No. 1). [file Data_Sheet_1.PDF]

# of frames | *k*-means selected | manually selected

128

256

512

1024

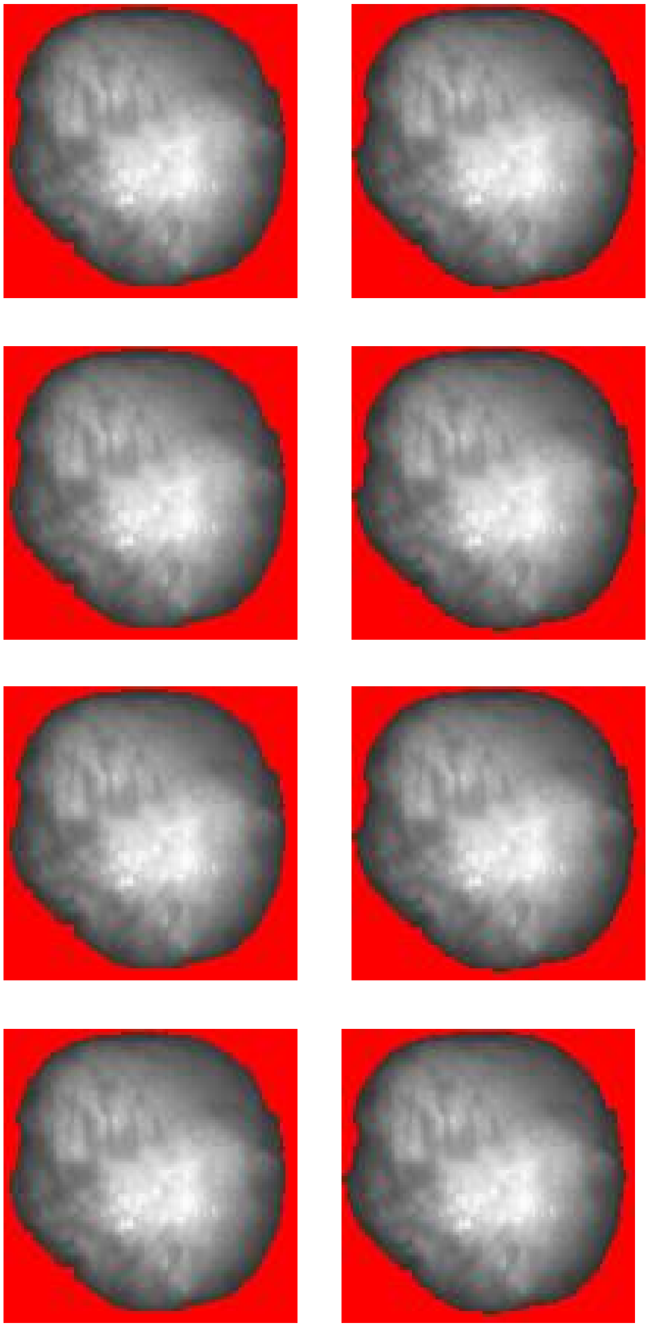

Figure S1

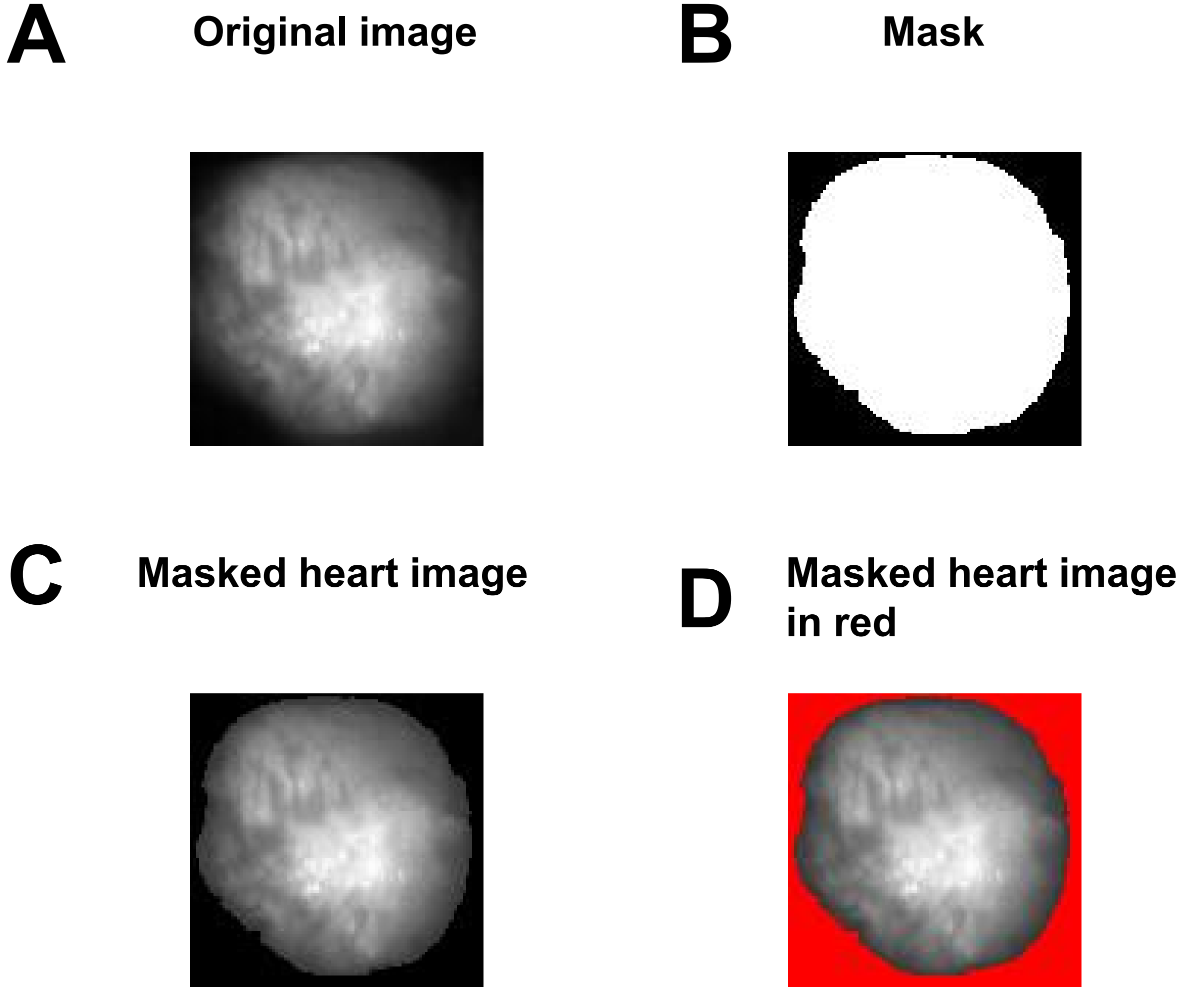
A
Original image
B
Mask
C
Masked heart image
D
Masked heart image in red

Figure S2

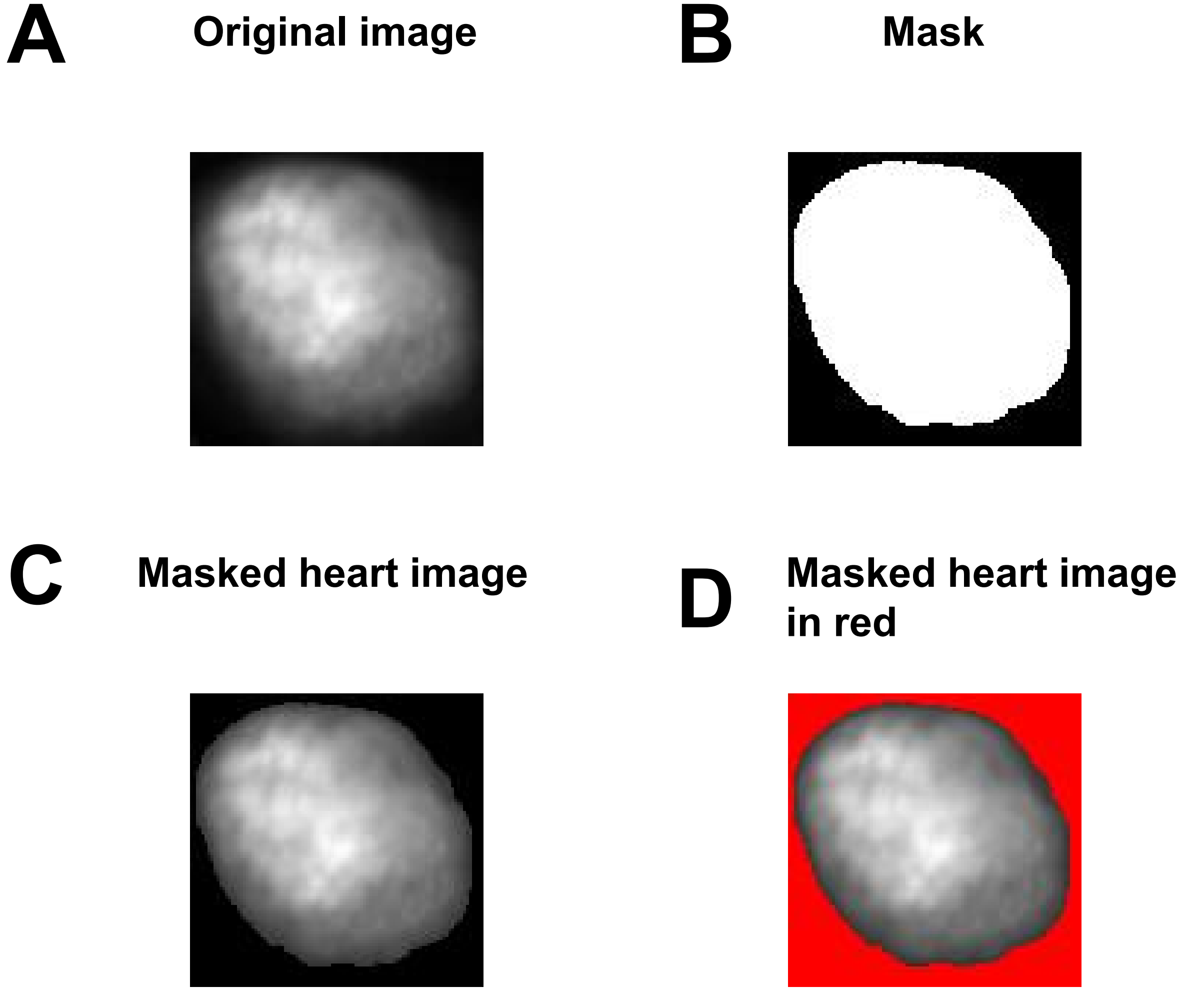
A
Original image
B
Mask
C
Masked heart image
D
Masked heart image in red

Figure S3

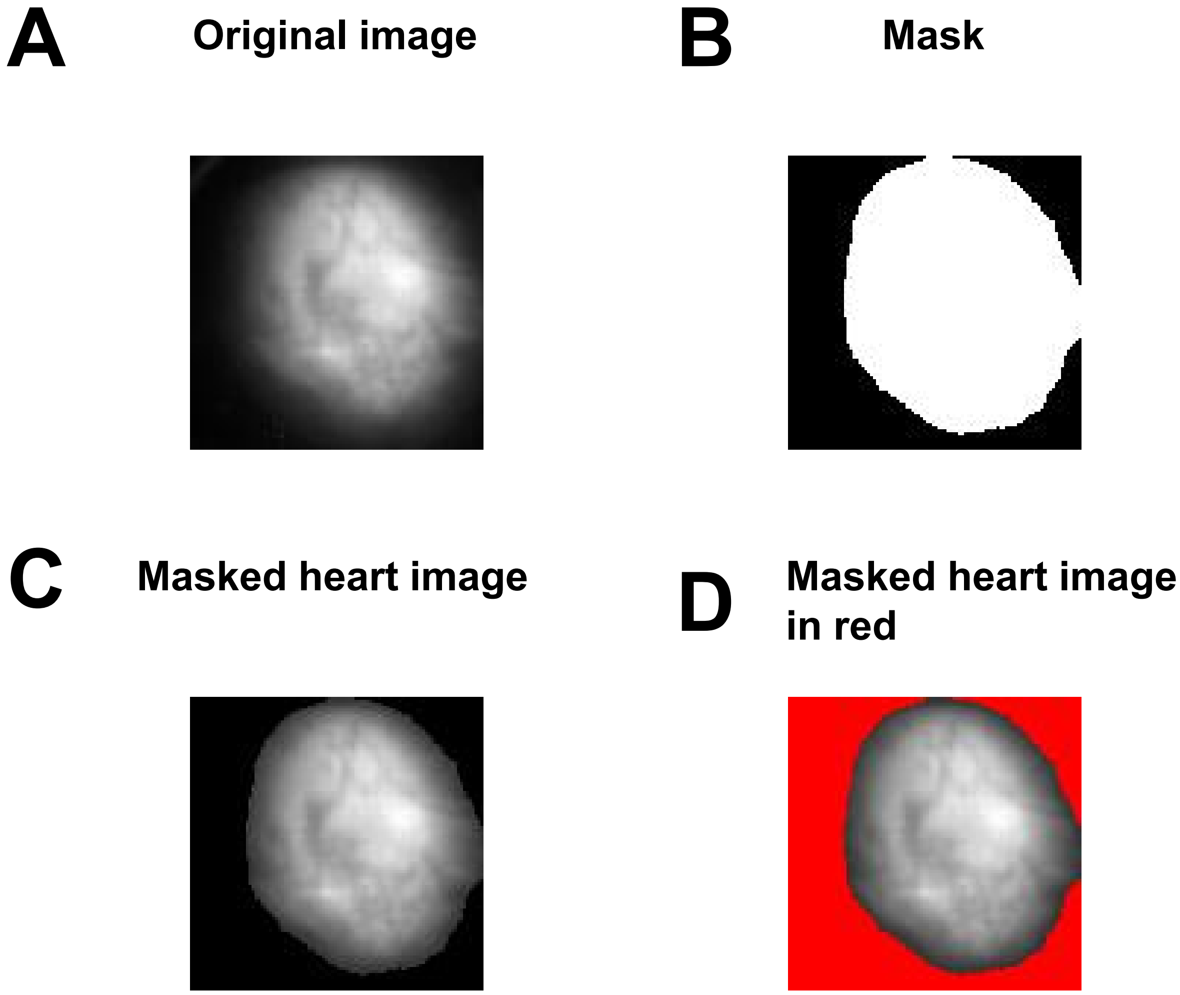
A
Original image
B
Mask
C
Masked heart image
D
Masked heart image in red

Figure S4

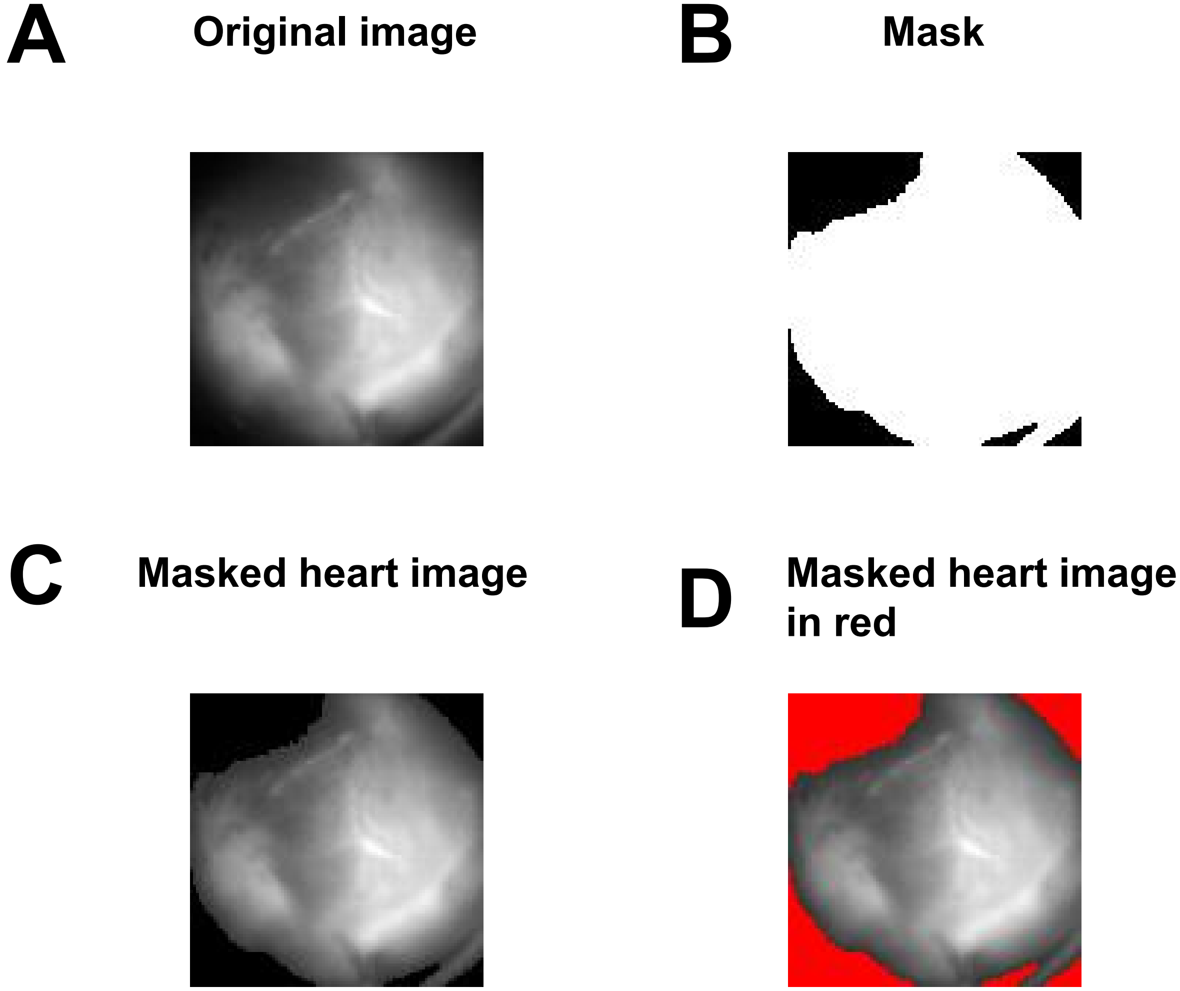
A
Original image
B
Mask
C
Masked heart image
D
Masked heart image
in red

Figure S5

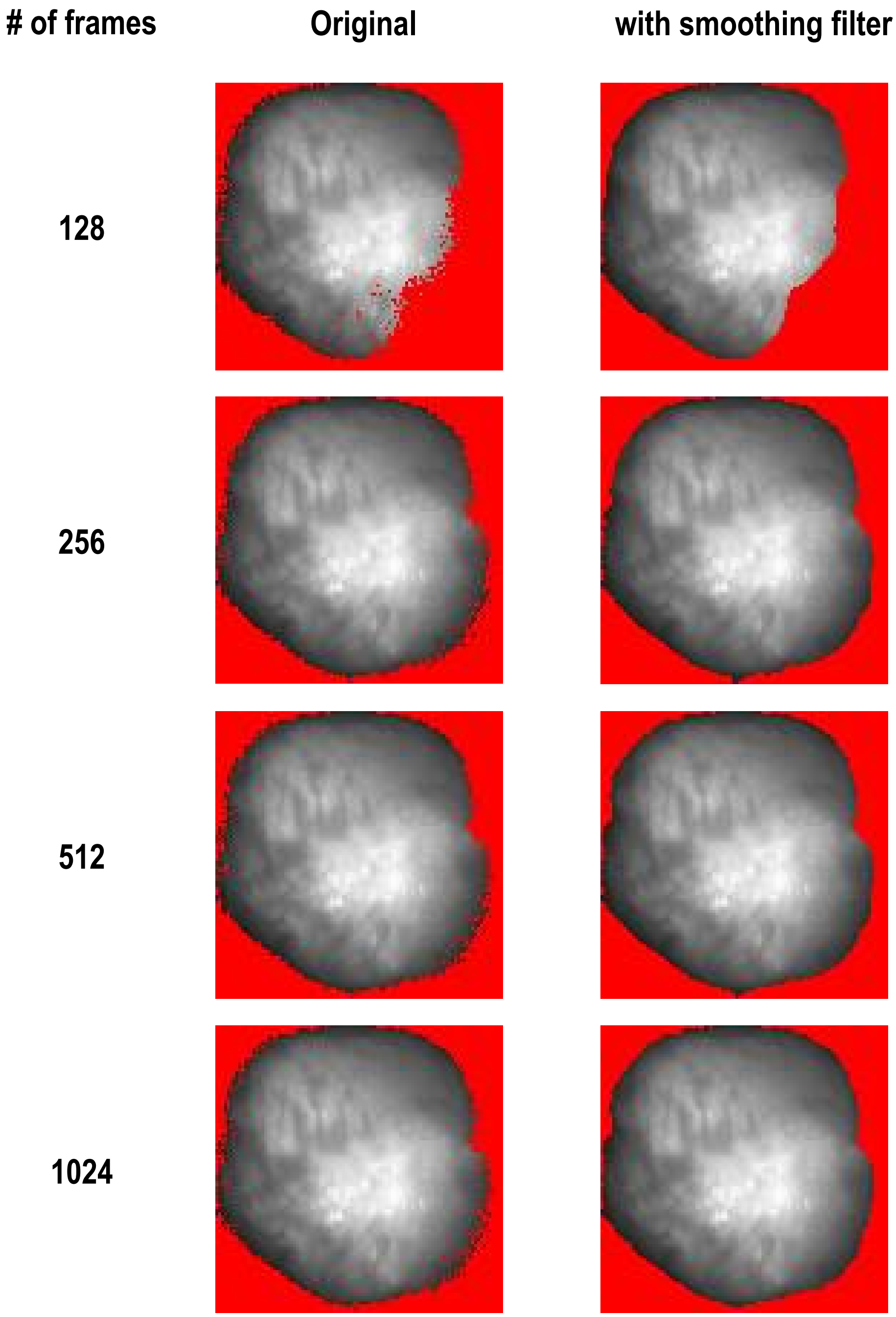

Figure S6

index of frames

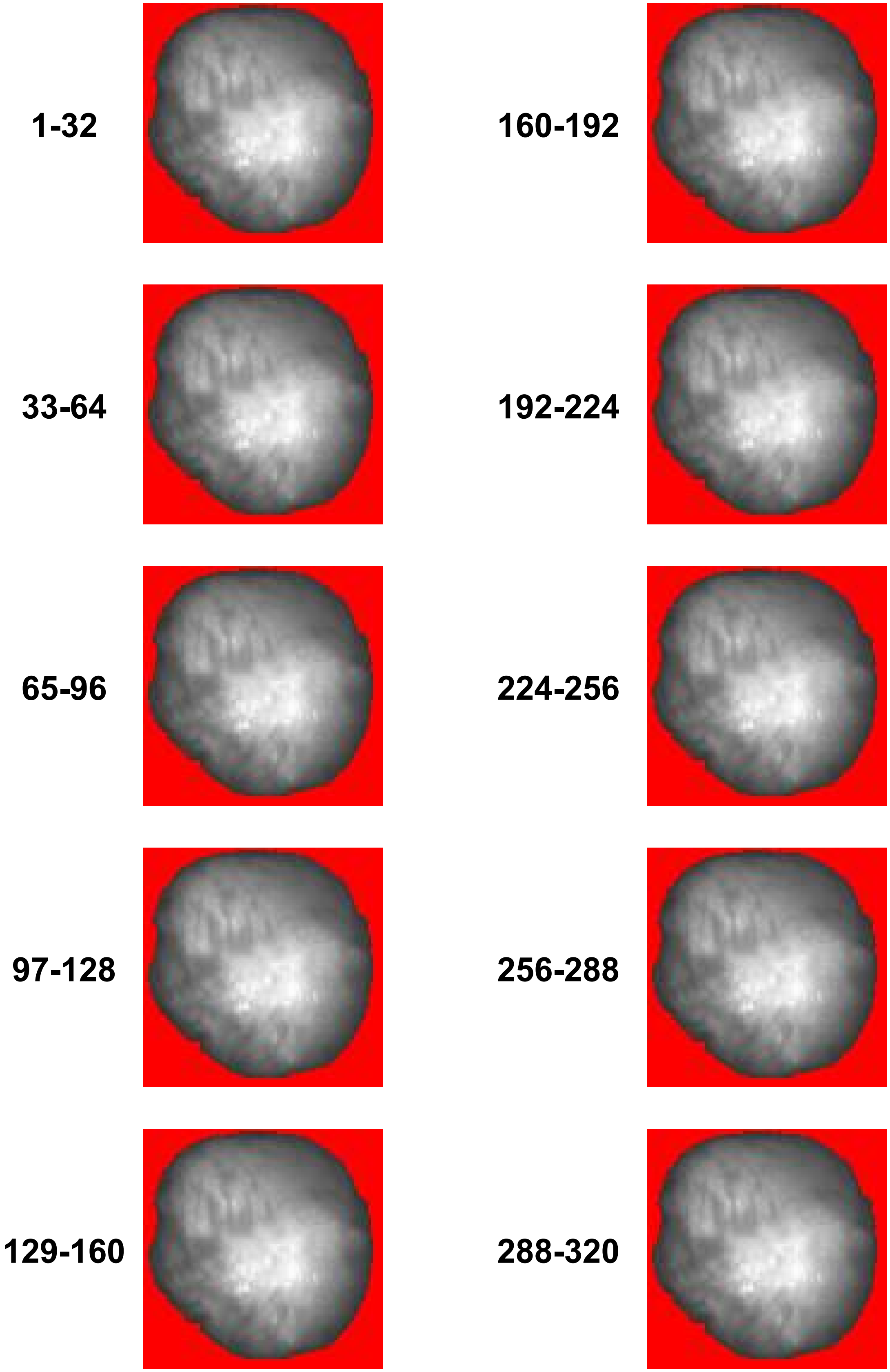

Figure S7

	128	256	512	1024	STD
k-means	0.627	0.740	0.742	0.740	0.0568
Logistic regression	0.712	0.731	0.718	0.474	0.1234
SVM	0.727	0.721	0.721	0.721	0.0030
Combined SVM	0.714	0.713	0.713	0.713	0.0005
Combined LR	0.709	0.733	0.743	0.808	0.04

Table S1
